# Migration of a nodule localisation marker to the contralateral lung

**DOI:** 10.1259/bjrcr.20210017

**Published:** 2021-05-26

**Authors:** Evangelos Skondras, Mohamed Basiony, Vladimir Anikin

**Affiliations:** 1 Department of Radiology, Harefield Hospital, Royal Brompton & Harefield NHS Trust, London, UK; 2 Department of Thoracic Surgery, Harefield Hospital, Royal Brompton & Harefield NHS Trust, London, UK; 3 Department of Oncology and Reconstructive Surgery, Sechenov First Moscow State Medical University, Moscow, Russia

## Abstract

Video-assisted thoracoscopic surgery (VATS) has been increasingly used to resect lung nodules avoiding thoracotomy thus reducing morbidity and hospitalisation time. One of the main challenges is to localise the target, because very often they are not palpable and small. Various nodule localisation techniques have been used to assist VATS resection including metallic marker implantation adjacent to the lesion of interest. These markers have been known to migrate, more often in the pleural space. We report an unusual case of metallic marker migration to the contralateral lung.

## Clinical presentation

An ex-smoker 70-year-old male with a history of COPD presented with repeated episodes of haemoptysis.

## Investigations

**Figure 1.**
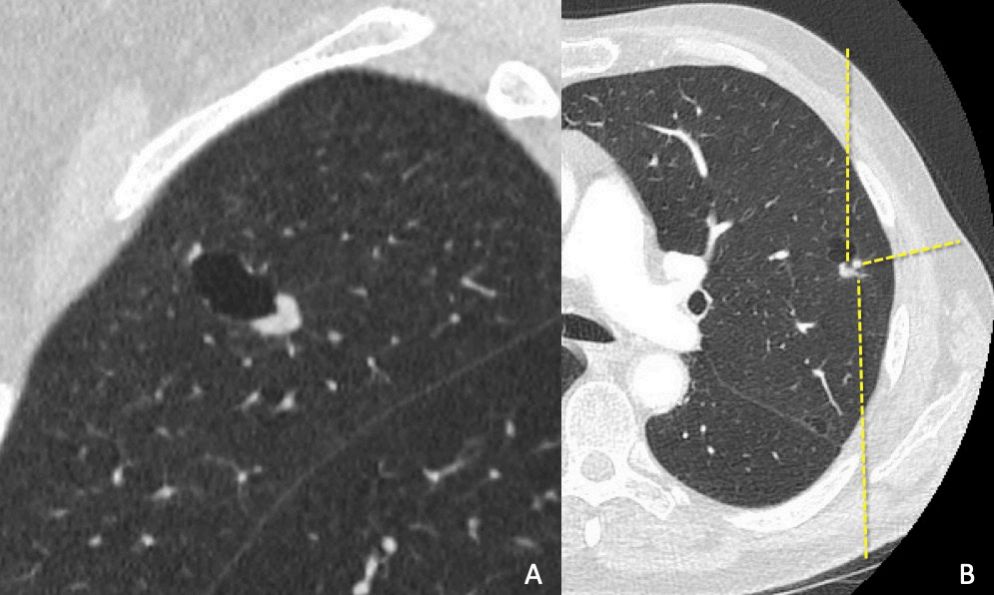
(A). A nodule with a cystic component in the left upper lobe seen in the pre-procedural CT scan. (B). Preprocedural planning with three possible marker stylet tracts (dotted lines)

A CT scan of the chest revealed a left upper lobe nodule with cystic component (Figure 1A), mildly FDG avid on PET-CT scan and suspicious for neoplasm.

## Treatment

Further preoperative assessment was done and a VATS resection was considered to be the most appropriate management.

Preoperative implantation of a localisation marker was deemed to be necessary for intraoperative localisation of the lesion.^1,2^ The radiology department in our hospital utilises 3-mm gold rods (Riverpoint Medical) preloaded in a 19G stylet as markers while the procedure takes place in the morning before the surgery under CT fluoroscopy guidance.

## Procedure

**Figure 2.**
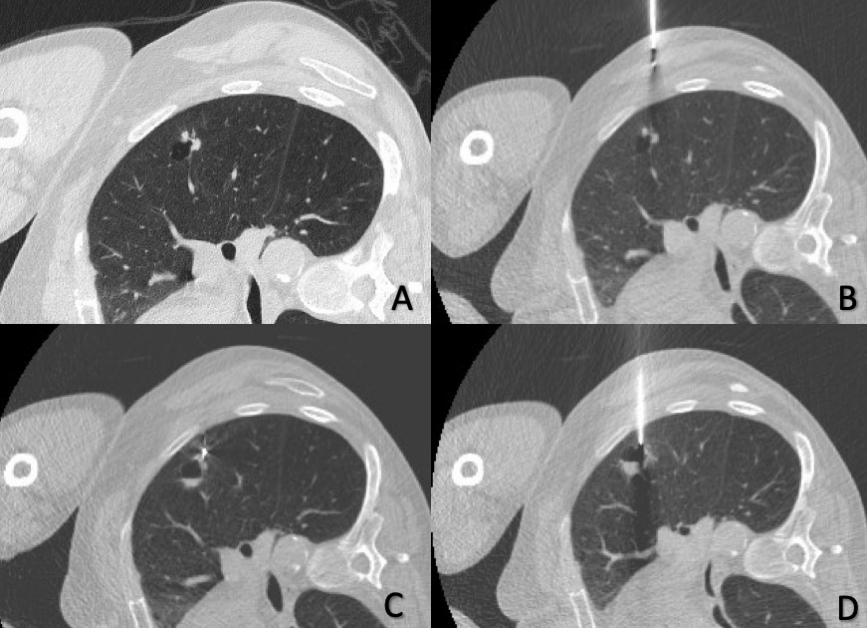
(A) and (B). Advancement of the preloaded stylet. (C). The marker is visualised adjacent to the solid component of the lesion. (D). Air fluid level is seen in the cystic component of the lesion. The marker is no longer visualised.

**Figure 3.**
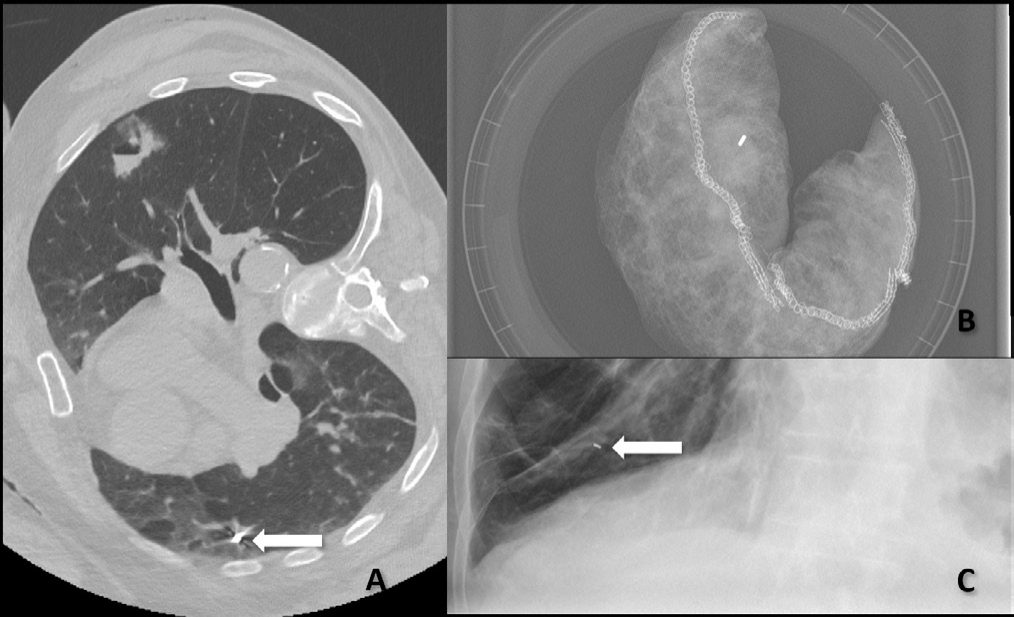
(A).Subsequent full coverage CT scan revealed migration of the marker to the dependant portion of the lateral segment of the right middle lobe. (B). Post resection radiograph confirmed the presence of the second marker within the resected specimen (C). Post-operative radiograph demonstrated the migrated marker in the right lower lung zone.

Pre-procedural CT scan of the chest revealed an unfavourable anterior needle access (through the cystic component) and difficult posterior access (Figure 1B). Therefore, the patient was placed in a right lateral recumbent position prior to the procedure. The preloaded stylet was advanced through the preselected path until the marker was visualised adjacent to the solid component of the lesion (Figure 2A, B and C). The patient experienced a short episode of coughing immediately after the marker was released. CT fluoroscopy revealed an air-fluid level within the cystic component of the nodule suggesting perforation from the stylet (Figure 2D) but the metallic marker was not evident. Unexpectedly, the subsequent full coverage CT scan of the chest revealed that the marker was resting in the dependent lateral segment of the right middle lobe (Figure 3A - arrow).

A second marker was successfully placed via the same route, and this time CT fluoroscopy confirmed correct deployment of the metallic marker adjacent to the left upper lobe solid nodule. The patient was then taken to the operating theatre and with a standard left VATS approach, the marker was identified based on the puncture within the visceral pleura. Left wedge resection was performed, and a radiograph of the specimen confirmed that the marker was present in the resected part of the lung (Figure 3B).

## Outcome and follow-up

Frozen section of the excised lung wedge showed focal granuloma and inflammatory tissue, and no further resection was undertaken. Subsequent paraffin section showed a non-mucinous adenocarcinoma, but the patient has chosen to undergo a follow-up rather then left upper completion lobectomy.

Postoperative radiographs showed interval stability in the location of the right lower lung zone marker without development of atelectasis (Figure 3C - arrow).

## Discussion

Percutaneously inserted lung localisation markers have been known to migrate most often in the pleural space especially if the lesion is subpleural.^3^ ^4,5^ More uncommon migration locations have also been reported^6^ . To our knowledge, this is the first report of contralateral lung marker migration in the literature.

The exact mechanism of the marker migration in this case is not clear. It is postulated that the combination of a lateral patient positioning and the accidental insertion of the marker within the cystic component of the nodule allowed for a gravitational intrabronchial “free-fall” of the marker via the major airways and the carina to the right middle lobe. It was not possible, however, to identify an airway communicating with the cystic component of the left upper lobe nodule and big enough to accommodate the 3 mm marker. An alternative explanation is that the entry needle overshoot and the marker was directly inserted within an airway of appropriate size although this was not evident in the CT fluoroscopy during the procedure.

Avoiding the lateral position probably would have prevented the marker migration but it was felt prior to the procedure that both the anterior and posterior access were unfavourable (Figure 1B). Another disadvantage of inserting a marker via the shortest needle path is marker migration within the pleural space.

Finally, marker implantation in a nodule with cystic component may lead to accidental insertion within an airway even if this communication cannot be appreciated in pre-procedural CT scan. Therefore, it is recommended that the operators should try and place the marker as far away from the cystic component as technically possible.

## Learning points

- Percutaneously inserted lung localisation markers can migrate within the lung parenchyma.
- Using a lateral approach for the insertion of lung markers can increase the likelihood of migration into the airways.
- If lateral approach is unavoidable, the patient should be quickly repositioned in supine or prone position after marker insertion to reduce the risk of migration.
- Marker insertion for a cystic nodule should be as far away as possible from the cystic component to reduce the risk of migration.
